# Anomaly Detection and Biomarkers Localization in Retinal Images

**DOI:** 10.3390/jcm13113093

**Published:** 2024-05-24

**Authors:** Liran Tiosano, Ron Abutbul, Rivkah Lender, Yahel Shwartz, Itay Chowers, Yedid Hoshen, Jaime Levy

**Affiliations:** 1 Department of Ophthalmology, Hadassah-Hebrew University Medical Center, Hadassah School of Medicine, Hebrew University, Jerusalem 9574409, Israellevjaime@gmail.com (J.L.); 2 School of Computer Science and Engineering, Hebrew University of Jerusalem, Jerusalem 9574409, Israel

**Keywords:** age-related macular degeneration, deep learning, anomaly detection, optical coherence tomography angiography

## Abstract

**Background**: To design a novel anomaly detection and localization approach using artificial intelligence methods using optical coherence tomography (OCT) scans for retinal diseases. **Methods**: High-resolution OCT scans from the publicly available Kaggle dataset and a local dataset were used by four state-of-the-art self-supervised frameworks. The backbone model of all the frameworks was a pre-trained convolutional neural network (CNN), which enabled the extraction of meaningful features from OCT images. Anomalous images included choroidal neovascularization (CNV), diabetic macular edema (DME), and the presence of drusen. Anomaly detectors were evaluated by commonly accepted performance metrics, including area under the receiver operating characteristic curve, F1 score, and accuracy. **Results**: A total of 25,315 high-resolution retinal OCT slabs were used for training. Test and validation sets consisted of 968 and 4000 slabs, respectively. The best performing across all anomaly detectors had an area under the receiver operating characteristic of 0.99. All frameworks were shown to achieve high performance and generalize well for the different retinal diseases. Heat maps were generated to visualize the quality of the frameworks’ ability to localize anomalous areas of the image. **Conclusions**: This study shows that with the use of pre-trained feature extractors, the frameworks tested can generalize to the domain of retinal OCT scans and achieve high image-level ROC-AUC scores. The localization results of these frameworks are promising and successfully capture areas that indicate the presence of retinal pathology. Moreover, such frameworks have the potential to uncover new biomarkers that are difficult for the human eye to detect. Frameworks for anomaly detection and localization can potentially be integrated into clinical decision support and automatic screening systems that will aid ophthalmologists in patient diagnosis, follow-up, and treatment design. This work establishes a solid basis for further development of automated anomaly detection frameworks for clinical use.

## 1. Introduction 

Retinal diseases affect millions of people worldwide, resulting in reduced visual acuity and blindness. Clinical diagnosis and follow-up exams rely extensively on optical coherence tomography (OCT) scans, which provide high-resolution, cross-sectional imaging of the retina layers [1]. Timely diagnosis is crucial for optimal treatment; however, at the earlier stages of the diseases, imaging biomarkers and lesions are more difficult to detect. Furthermore, due to the subjective nature of the task, often there is a disagreement even among domain experts when describing the features in retinal scans. Therefore, there is a need for an automated clinical decision support system that will aid ophthalmologists in the early detection of biomarkers for the development and progression of the disease.

Anomaly detection (AD) is a subclass of machine learning methods based on a binary classification between normal and anomalous classes. Although visual anomaly detection is highly valuable, it also poses significant challenges. One major challenge is that it is not always possible to train a model with full supervision for a given task due to the wide variety of anomalies and the lack of classified anomalous data. Hence, retinal datasets often contain annotation at the disease stage, but not particularly for biomarkers and lesion levels. The field of image anomaly detection has transformed in recent years due to the emergence of deep networks [2]. The strength of these networks lies in their ability to learn meaningful representations of images. Similar semantic images are typically assigned close numerical representation. In contrast, raw image pixels provide a much weaker representation. The latest state-of-the-art anomaly detection methods use a simple three-step paradigm: (1) compute a numerical representation for each image in the normal dataset using a deep neural network; (2) estimate the normal data distribution with a probabilistic model; (3) calculate the likelihood for every test sample employing the probabilistic model [2]. If the model considers an image unlikely, it is labeled anomalous. Despite training on non-medical datasets, these methods performed well on retinal images. Burlina et al. validated that such methods achieve state-of-the-art performance for retinal fundus images [3]. Retinal pathologies such as choroidal neovascularization (CNV), diabetic macular edema (DME), and drusen create a diverse set of anomalous images. CNV severely disrupts the retinal structure, DME results in swelling of the retinal layers, and drusen appears as localized elevation of the retina and is the hallmark of age-related macular degeneration (AMD), the leading cause of blindness in the elderly population in the Western world.

Thus, the aim of this study was to identify those common retinal pathologies using novel self-supervised anomaly detection deep learning methods.

## 2. Methods

This retrospective study used the Kaggle dataset, a public domain dataset of 84,495 OCT slabs (Spectralis OCT, Heidelberg Engineering, Heidelberg, Germany) from 44,346 individuals and a second local real-world dataset—the Hadassah Imaging Dataset—which consists of OCT volume scans (Spectralis OCT, Heidelberg Engineering, Heidelberg, Germany) of 348,657 OCT scans from 4663 patients diagnosed with age-related macular degeneration. Four categories were used for the model training: normal retinal structure, CNV, DME, and early or intermediate AMD with the presence of drusen. The study adhered to the tenets of the Declaration of Helsinki. Institutional review board (IRB) approval was obtained from the Hadassah Medical Center (0382-19). As this was a retrospective data collection study, a waiver of informed consent was granted. The dataset was divided into training, validation, and test sets. We used the test and validation sets for evaluation. In addition, we used a validation set as suggested in Sparse-GAN [4]. The validation set comprised 4000 normal and 1000 anomalous image slabs that were randomly chosen from each of the categories. This dataset presents a challenge for anomaly detection as the retinal scans are not aligned, not centered, and contain significant noise. Additionally, and most challenging, the anomalies are diverse in their characteristics and location through the different layers of the retina.

Our novel approach follows the latest state-of-the-art (SOTA) methods paradigm and utilizes local region representations of healthy scans. We first employ the pre-trained neural network over a healthy retinal scans dataset to obtain a local representation bank. Then, we fit a probabilistic model for the local representations. The probabilistic model was applied to new OCT scans to estimate the likelihood of their local regions. Local regions with low likelihood scores were deemed abnormal. Finally, retinal scans that consist of one or more abnormal regions were classified as anomalous. Additionally, we used the regions’ likelihood scores for the localization of anomalous patches. An initial self-supervised fine-tuning step could be performed on the pre-trained neural network. This step adapts the neural network to the normal retinal dataset and enhances the method’s performance. We use center loss adaptation, as suggested in PANDA, to fine-tune the pre-trained neural network and achieve better results [2].

We compared four SOTA frameworks for anomaly detection and segmentation in images. All the frameworks in this work used pre-trained CNNs for feature extraction and embedding creation. The four SOTA frameworks are: (1) Pretrained anomaly detection adaptation (PANDA) [2]. This framework consists of several stages: (i) Feature extraction using a pre-trained deep neural network and adaptation, (ii) retrieval of the nearest K normal neighbors to the target, and calculation of anomaly scoring. (2) Semantic Pyramid Anomaly Detection (SPADE) [5]. This framework consists of several stages: (i) Image embedding extraction using a pre-trained deep neural network, (ii) retrieval of the nearest K normal neighbors (kNN) to the target, and (iii) calculation of dense pixel level correlation between the target and the normal images. (3) Patch Distribution Modeling (PaDiM) framework [6]. Stages included (i) Image embedding extraction using a pre-trained deep neural network, (ii) fitting a multivariate Gaussian distribution to every patch using the normal image embedding, and (iii) estimation of the plausibility of every patch in the target image using the Mahalanobis distance between the target patches and the corresponding multivariate Gaussian distribution. (4) PatchCore framework [7]. This framework consists of several stages: (i) Creating patch embedding for every image in the training set, (ii) sub-sampling the patch embeddings using the coreset mechanism, (iii) calculating segmentation maps and image-level anomaly scores for each sample from the test set using the normal images patch embeddings sub-set.

Implementation details: an initial preprocessing stage included black-and-white margin removal using classical image processing algorithms. Specifically, a contour detector was applied to find white and black margins. In cases where the corners were part of a contour, we checked if each row or column held more than a quarter of white or black pixels and removed them in such cases. Then, the images were resized to 224 × 224. ResNet152 pre-trained on ImageNet 1 k was used as a deep neural network for the representation extraction [8]. We evaluated both local region and image-level representation methods. We represent each local region by combining the features of the second and third residual blocks of the ResNet152 model. In addition, the features after global average pooling are used as image-level representations. K nearest neighbors were utilized for the density estimation with k = 5 [9]. For the local region approach, the density estimation takes a global approach. A local region’s density was estimated against all local regions within the nominal bank, regardless of their spatial location. In order to reduce the size of the local region bank and lower the computational cost, we used a greedy coreset. The nominal bank keeps 1% of healthy local regions for the open source and 10% for the clinical dataset. In the fine-tuning step, we used PANDA for feature adaptation over the training data for 40 epochs [2].

After training the model on normal-only training OCT scans, a nominal local bank was extracted. The model was operated to score each of the OCT scans in the test set using kNN. The ROC AUC was used as a parameter-free criterion for scoring the model. Every experiment was conducted with 3 different random seeds. All studies were performed on NVIDIA RTX 2080 GPU cards using Pytorch 2.3.0.

## 3. Local Retinal Region Anomaly Detection

**Figure 1:**
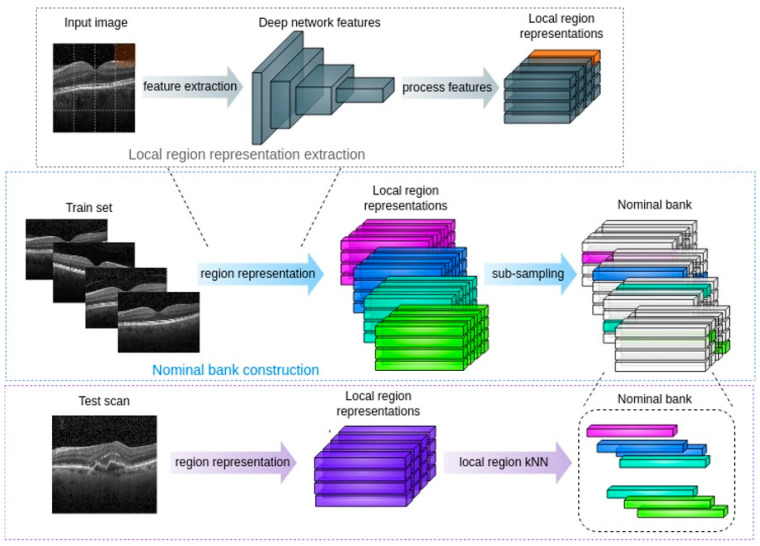
Demonstration of local region anomaly detection workflow. The non-medical pre-trained neural network learns local region representations. Regions with similar properties are mapped to numerically similar representations, while dissimilar local retinal regions are mapped to dissimilar representations. The last row presents a test scan with choroidal neovascularization which the fluid representation differs from the present of the pigment epithelial detachment and drusen.

Data representation: numerical representations of local regions are extracted using a deep neural network from healthy retinal scans. The neural network is pre-trained on non-medical images but has been shown to learn effective representation for medical images, including color fundus photos [10,11,12]. The representations need to satisfy the following property: regions with similar properties should be mapped to numerically similar representations, while dissimilar local retinal regions should be mapped to dissimilar numerical representations. For example, OCT regions that contain drusen should map to the same deep representation, which should differ from fluid-presented OCT representation (Figure 1).

Density estimation: following representation extraction, we estimate the distribution of nominal retinal regions. To represent the normal data, we train a probabilistic model. Models typically fall under two categories: parametric (following a simple, mathematically specified function) and non-parametric models. Unfortunately, the appearance of local retinal regions is complex and highly variable and, therefore, does not fit a simple mathematical form. Instead, we opted for a well-known non-parametric model, the K nearest neighbors (kNN) distance [9]. For a target image, the model specifies the likelihood of a region by first retrieving the K nominal local region with the most similar representation using the nominal region bank. We then record the average of the distances between the numerical representation of the target local region and each of the representations of the k nearest local regions. The kNN distance measures the likelihood of the local region being anomalous. Regions with high kNN distance are very atypical, i.e., their representation is not like the representation of any previously observed normal region. These regions have a very low likelihood and are classified as anomalous. Conversely, representations of regions with low kNN distance are similar to those observed as normal; hence, these regions are expected to be normal. Note that the kNN distance is not a calculation of probability calculation [13,14]. However, their results are highly correlated to kNN results, and the kNN has less computational starving.

Efficient nearest neighbors: we reduce the number of healthy local regions in the nominal bank to make the similarity search more efficient. The computation of the kNN can be slow for large datasets due to neighbor retrieval. We encounter the same case in retinal localization, where each training image consists of many local retinal regions. The train set contains thousands of normal scans that enlarge the amount of local retinal regions. The likelihood is estimated over local regions and may be computation-consuming. In order to speed up the computation of the kNN, most approaches typically attempt to reduce the number of potential matches without removing the likely matches. We follow previous methods in choosing a reduced number of prototypical regions that provide a compressed representation of all the nominal regions observed during training. The prominent approaches for performing this compression are K means and coreset methods, and in this work, we followed PatchCore in using an approximation algorithm of the core set for normal region dataset compression [7,9].

Representation fine-tuning on retinal data: neural networks pre-trained on external non-medical datasets extract powerful representations even for ophthalmic data. They may be further improved by another training stage on the healthy retinal scan data. We employ the deep network and extract image-level representations for the healthy training set. Then, we compute the center of the numerical representations using their average. A center loss is used to fine-tune the network over the healthy scans. It demands that the representations of normal scans would be close to the calculated center. As a result, we hoped that the normal scan representations would be closer to the center than the anomalous scans. To prevent catastrophic forgetting, we include an elastic weight consolidation regularization loss in addition to representation center loss as advocated by PANDA [2].

Full retinal scan anomaly detection: after extracting anomaly scores for each local region of a retinal scan, the next step was to classify the scan as normal or abnormal. Our approach defines the anomaly score as the maximum of the anomaly scores of all local regions within the retinal scan. The underlying logic was that an anomaly is of interest even if it lies in a small region of the retina. Such an approach can help detect even small lesions and changes in the retina structure.

## 4. Results

The study was designed to answer several questions: (i) Should anomaly detection be performed at the local region or image level? (ii) Are representations learned using non-medical images effective for anomaly detection on OCT retinal scans? (iii) Given the effectiveness of pre-trained features, are there benefits to self-supervised training over the normal data? (iv) Can inter-scan alignment assumptions be used in the nearest neighbor procedure? (v) Are the local region anomaly scores informative for humans?

The local region approach had generally greater accuracy, sensitivity, specificity and F1 score comparing the tested SOTA methods, with averages of 94.8, 94.4, 95.9, and 0.95, respectively. Table 1 presents the comparison between the different methods.

## 5. Image-Level vs. Local Region Representation

In the approach presented by Burlina et al. [3]. Anomaly scoring is performed using image-level features. A representation is computed for the entire retinal scan, and an anomaly score is assigned to the image based on the extracted representation. In contrast, we advocate a local approach, which scores regions rather than the entire retinal scan. Local region representations were computed using a deep network followed by a region anomaly scoring stage. The set of region scores is used to compute an image-level anomaly score. The two approaches are compared in Table 2.

## 6. Self-Supervised vs. Auxiliary Pre-Trained Feature Representations

We compare two approaches for learning representations. The first is self-supervised representation learning, which only uses the training data consisting of healthy OCT retinal scans. The second approach uses a feature representation trained on an auxiliary, non-medical dataset—ImageNet. Note that the ImageNet dataset contains a label for every image indicating its object category (e.g., dog, cat, car, etc.). ImageNet images are very different from OCT scans, apart from the obvious difference of not containing retinas, they also consist of color images and are relatively noise-free. The comparison between the approaches is presented in Table 3. We can observe that ImageNet-pretrained representations outperform the self-supervised representation. This is non-trivial, as during training, the model does not observe any retinal OCT scans.

## 7. Representation Fine-Tuning

Although representations pre-trained on non-retinal images are clearly effective for retinal OCT anomaly detection, it begs the question of whether incorporating retinal scans into the training images will result in further improvements. To test this hypothesis, we fine-tuned the ImageNet-pre-trained representation on the training set, which consists of healthy OCT scans. Specifically, we used the procedure introduced in PANDA [2]. We present the results in Table 4. It can be observed that the proposed adaptation stage improved both image-level and local-level anomaly detection approaches. The performance is particularly significant for OCT scans containing drusen. This demonstrates that using both auxiliary non-retinal data as well as the normal OCT scans provided for training is more effective than merely the auxiliary pre-training dataset.

## 8. Slice Alignment Assumptions

Previous local region anomaly detection approaches (e.g., PaDiM) have suggested that the anomaly score for each local region may be computed by comparing its representation to those of other regions having the same spatial location in the image [6]. In other words, a region centered around the (x, y) pixel location is compared with other regions across different slices and scans that are also centered around the (x, y) pixel. This assumes that different scans are aligned. This approach has multiple benefits when the slices are aligned, including faster runtime and higher accuracy. However, these advantages are only beneficial when the scans are indeed well aligned. This approach was compared to the standard, global approach of comparing all local regions regardless of their center location. The results are presented in Table 5. We can observe that the alignment assumption is not effective for unaligned retinal scans and results in inferior performance relative to the global search.

## 9. Discussion

In this paper, we present a novel approach for anomaly detection for retinal OCT scans. Our approach is based on local region anomaly detection and localization. First, a numerical representation was computed for each local region of the retinal scan. We then fit a model for the local representations—estimating the likelihood that each local region of the retinal scan is normal. We used this model to localize the normal and anomalous regions of the retina scans. Finally, retinal scans that contained one or more anomalous regions were classified as anomalous. Furthermore, our method represents data using a neural network that was pre-trained on an external, non-medical dataset. Our method was shown to perform well on two datasets, a popular public dataset as well as a local real-world clinical dataset.

Anomaly detection and localization are vital fields in visual machine learning. The methods in the fields can be categorized into several categories: reconstruction-based, distribution-based, and classification-based. 

Reconstruction-based methods learn to reconstruct the normal training data using a set of basis functions. During the reconstruction process, these methods use a bottleneck to create a compressed representation, which is, in turn, used to reconstruct the input sample. The main idea is that normal samples should be reconstructed accurately, while anomalous data will not. Therefore, the reconstruction loss is used as the anomaly criterion. Classical reconstruction-based methods are k nearest neighbors (kNN) [9], K-means [15], and principal component analysis (PCA). In recent years, the use of deep learning methods has expanded to the field of reconstruction-based methods. Deep learning architectures such as Auto-Encoder (AE) [4,16], Variational Auto-Encoder (VAE) [17], and Generative Adversarial Networks (GAN) [18] are all trained to reconstruct normal images. The common reconstruction loss functions are Euclidean distance, L1, and structural similarity (SSIM) [14]. Deep learning architectures also use deep perceptual loss and discriminators as adversarial loss functions [13].

Distribution-based methods attempt to learn the probability density function (PDF) of the distribution of normal samples. The anomaly score for every given image from the test set is calculated by the PDF. The expectation is that normal images will be mapped to dense areas using the PDF so that their likelihood will be low compared to anomalous images. Therefore, in cases where the anomaly score of an image is larger than a given threshold, the image is deemed anomalous. Distribution-based methods differ in the distributional assumptions that they adopt. Such methods include parametric models such as multivariate Gaussian and Gaussian mixture models and non-parametric models such as kernel density estimation, robust kernel density estimation, and KNN, which also performs density estimation [5,6].

There is a known difficulty in achieving good results with this approach when handling high-dimensional data due to the PDF estimation. To mitigate this problem, deep learning methods are used to project the high-dimensional data to lower-dimensional space. Works such as PANDA [5], SPADE [6], and PatchCore [7] use pre-trained CNNs to extract embedding and use kNN to evaluate the anomaly score for a sample in the test set. Other approaches use AE or GAN architectures, such as DAGMM, which employs a combination of distribution and reconstruction loss [19].

Classification-based methods are another paradigm for anomaly detection. These methods find a separate manifold between the normal and the anomalous data in a given representation space. Such methods are one-class support vector machines (OC-SVM) and their variants, such as support vector data description (SVDD), which obtain a spherically shaped boundary around a given dataset. Recently, deep learning methods have been used to improve classical approaches. Examples of such methods are Deep SVDD and deep OC-SVM. An additional set of deep learning methods uses the self-supervised training approach, in which models attempt to solve an auxiliary task with either pre-existing or easily obtained data. Golan and El-Yaniv devised the Rot-Net-based method, a self-supervised method that utilizes rotation transformations to classify test samples. The method was later improved and expanded [7,20].

**Figure 2:**
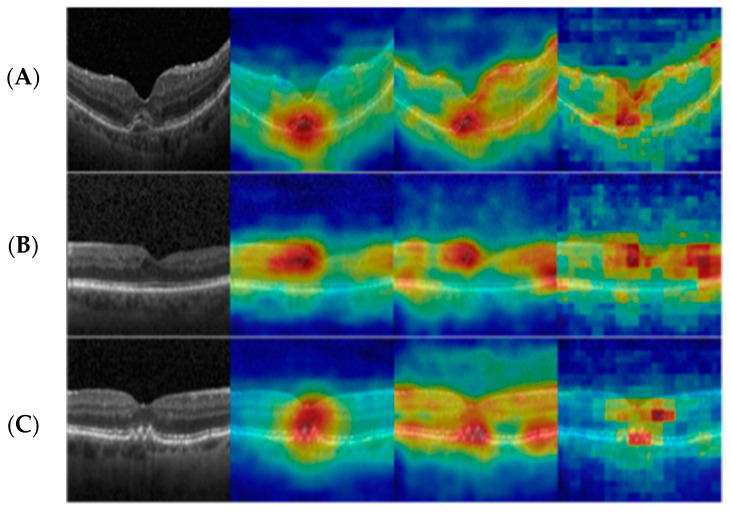
Heat maps of anomalous retinal OCT scans containing (**A**) choroidal neovascularization, (**B**) diabetic macular edema, and (**C**) drusen. Column 1 presents the original OCT scan. Heat-maps were extracted by local region using aligned density estimation (column 2) and local region with global density estimation (column 3).

Another unique challenge that visual anomaly detection presents is anomaly localization. For every detected anomalous scan, the anomaly detection algorithm segments all the areas that were identified as anomalous. This setting is important for algorithm explainability (Figure 2), as it sheds light on the algorithm’s decision. Additionally, in the field of medical images, anomaly segmentation can help detect disease biomarkers and lesions in the scans. These algorithms have the potential to reveal new biomarkers and thereby improve diagnosis. This might also be a significant step in evaluating the clinical-pathological correlation between known or new biomarkers and visual acuity.

Drusen, for example, is the hallmark of AMD which represents pathological proteinaceous and lipids accumulation between the choroid and the retinal pigment epithelium.

Drusen is supposed to be a pathogenic trigger of the disorder. In fact, drusen may mechanically alter retinal function. A novel hypothesis exists, suggesting that a metabolic defect (systemic or focal within the retinal pigment epithelium) may be the real determinant of visual impairment while causing the concomitant accumulation of the drusen. Pinelli et al. did not find a clear correlation between visual impairment and the occurrence of drusen number, size, and the extent of a drusenoid area in the foveal region in 120 AMD patients. The authors concluded that drusen and visual deterioration develop because of similar upstream biochemical alterations, but it is likely that drusen by itself does not produce visual deterioration [21]. Accurate localization of biomarkers like drusen and its geometry features, especially in the macular cube using AD algorithms, might obtain insight into analyzing a quantitative correlation between drusen (or other anomalous regions) and visual impairment in a specific macular zone.

Anomaly segmentation has received less attention compared to image-level anomaly detection. Early works proposed patch-based localization using classical approaches [22,23]. Later works expanded these patch-based methods and used pre-trained CNNs as a feature extractor for a K-means classifier [24]. Bergmann et al. introduced the MVTec dataset and evaluated GAN, AE, and classical methods with this dataset. Bergmann et al. followed up on this work with a student-teacher approach, which uses a pre-trained CNN [25]. Venkataramanan et al. introduced an attention-guided anomaly localization framework that uses VAE and other methods, such as GradCAM and adversarial loss [26]. Recently, a number of distribution-based frameworks achieved SOTA performance in the task of anomaly localization, namely PANDA, SPADE, PaDiM, and PatchCore. These frameworks utilize pre-trained CNNs for feature extraction and embedding creation and use density estimations for anomaly scoring.

In this work, four SOTA frameworks for visual anomaly detection were adapted to the domain of retinal OCT scans. Anomaly detection and localization frameworks have the potential to detect disease biomarkers and lesions, creating the ability to diagnose retinal pathologies using OCT scans. We showed that with the use of pre-trained feature extractors, these frameworks can generalize to the domain of retinal OCT scans and achieve high image-level ROCAUC scores. Additionally, adapting the feature extractor to the OCT scan domain can cause the framework to outperform the “raw” baseline. The localization results of these frameworks are promising and successfully capture areas that indicate the presence of retinal pathology. In this research, we have evaluated OCT sections obtained from eyes affected by AMD and DME, which comprise the most common cause of blindness in older people and in the working age group, respectively. There are multiple other sigh-threatening retinal pathologies, and the fact that such unsupervised anomaly detection frameworks obtained clinical-level performance suggests that other retinal pathologies may also be readily identified in OCT sections. Such frameworks for anomaly detection and localization can be integrated into clinical decision support systems that will aid ophthalmologists in patient screening, diagnosis, follow-up, and treatment design.

Caveats of our study include a relatively lower number of scans and different image quality parameters like different scan numbers in each B-scan volume, different ART values, and different alignment of the image. Yet, the OCT images studied where from a commonly used OCT devise which produces high-quality sections. Furthermore, we have applied an unsupervised approach, which reduces the risk of bias generated by training.

This preliminary work establishes a solid basis for further development of automated anomaly detection frameworks for clinical use. Future works should focus on exploiting information that exists beyond a given OCT scan, such as diagnosis or patient medical history, to improve the clinical performance of the automatic anomalous detection algorithm. This direction has great potential for groundbreaking results with far-reaching implications.

## Figures and Tables

**Table 1:** Quantitative comparison with the state-of-the-art methods.

	Validation Set		Local Region	
	AUC	Sensitivity	AUC	Sensitivity
Auto-Encoder	72.9	78.3	75.1	83.4
AnoGAN	81.5	84.6	78.9	91.7
F-AnoGAN	84.9	88.2	80.8	87.1
Pix2pix	83.7	87.4	81.5	90
Sparse-GAN	88.5	92.5	84.1	95.1
Local region	98.6	99.7	97.8	98.1

AUC: area under the curve, GAN: Generative Adversarial Networks.

**Table 2:** Comparison of local region and image-level anomaly detection methods (ROC AUC%).

	Validation Set		Test Set	
	Image-Level	Local-Level	Image-Level	Local-Level
CNV	98.7	99.9	99.4	100
DME	95.4	99.1	97.9	100
Drusen	92.1	96.7	93.6	99
Average	95.4	98.6	97	99.7

AUC: area under the curve, CNV: choroidal neovascularization, DME: diabetic macular edema.

**Table 3:** Comparison of pre-trained and self-supervised (CutPaste) features ROC AUC%.

	Validation Set		Test Set	
	Self-Supervised	Pre-Trained	Self-Supervised	Pre-Trained
CNV	96.7	99.8	97.4	100
DME	96.7	98.9	98.6	100
Drusen	81.9	97	82.1	99.1
Average	91.8	98.6	92.7	99.7

CNV: choroidal neovascularization, DME: diabetic macular edema.

**Table 4:** An evaluation of feature adaptation (ROC AUC%).

	Image-Level		Local-Level	
	Pre-Trained	Adapted	Pre-Trained	Adapted
CNV	98.7	99.1	99.8	100
DME	95.4	95.9	98.9	99.9
Drusen	92.1	93	97	99.4
Average	95.4	96	98.6	99.8

CNV: choroidal neovascularization, DME: diabetic macular edema, AUC: area under the curve.

**Table 5:** Evaluation of the alignment assumption ROC AUC%.

	Aligned	Global
CNV	84.3	98.3
DME	85.1	93.7
Drusen	74.2	86.7
Average	81.2	92.9

CNV: choroidal neovascularization, DME: diabetic macular edema.

## Data Availability

The data presented in this study are available on request from the corresponding author.
